# Papillary carcinoma arising from a thyroglossal duct cyst

**DOI:** 10.4103/0971-3026.50828

**Published:** 2009-05

**Authors:** S Smiti, Nabil Sherif Mahmood

**Affiliations:** Department of Radiodiagnosis, Kasturba Medical College, Manipal-576 104, Karnataka, India

**Keywords:** Carcinoma, papillary, thyroglossal cyst

## Abstract

This report describes a case of papillary carcinoma arising from a thyroglossal duct cyst (TDC) in a young woman. Imaging showed a heterogeneous cystic lesion at the level of the hyoid, with calcifications and enhancing septae. We compared the USG, CT scan, and MRI findings with those reported previously in literature and we conclude that the presence of a midline cystic lesion with calcification in a young adult should arouse suspicion of papillary carcinoma in a TDC.

Thyroglossal duct cyst (TDC) is the most common developmental cyst in the neck and is commonly located in the infrahyoid region. Carcinoma arising from a TDC is very rare and is detected per-operatively in most cases.[1] Preoperative imaging plays an important role in differentiating benign from malignant TDC and in planning surgical management.

## Case Report

**Figure 1:**
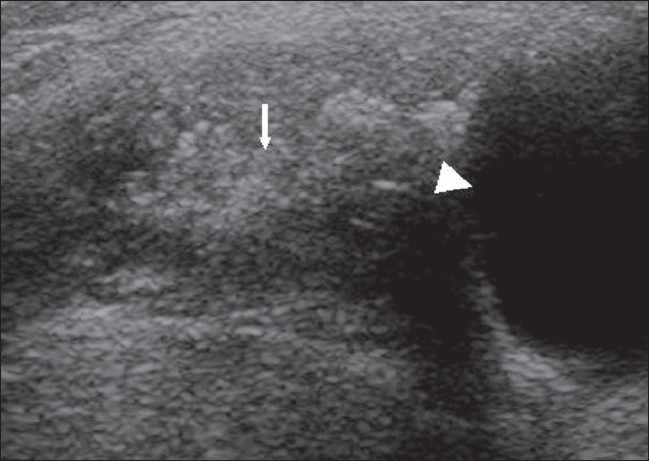
USG at the level of the hyoid bone shows a cystic lesion (arrowhead) with a heterogenous component containing calcification (arrow)

**Figure 2(A, B):**
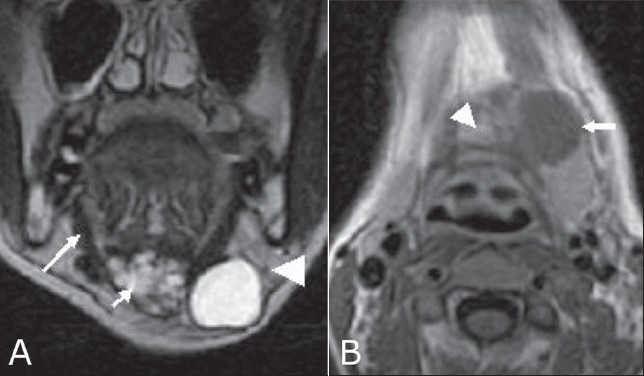
Coronal T2W (A) image shows a hyperintense multicystic lesion (small arrow) extending into the floor of the mouth between the bellies of the mylohyoid (large arrow). The large cyst abuts the left submandibular gland (arrowhead). Axial post-contrast T1W image (B) shows an enhancing, midline, cystic component (arrowhead) adjacent to the left paramedian cyst (arrow)

**Figure 3:**
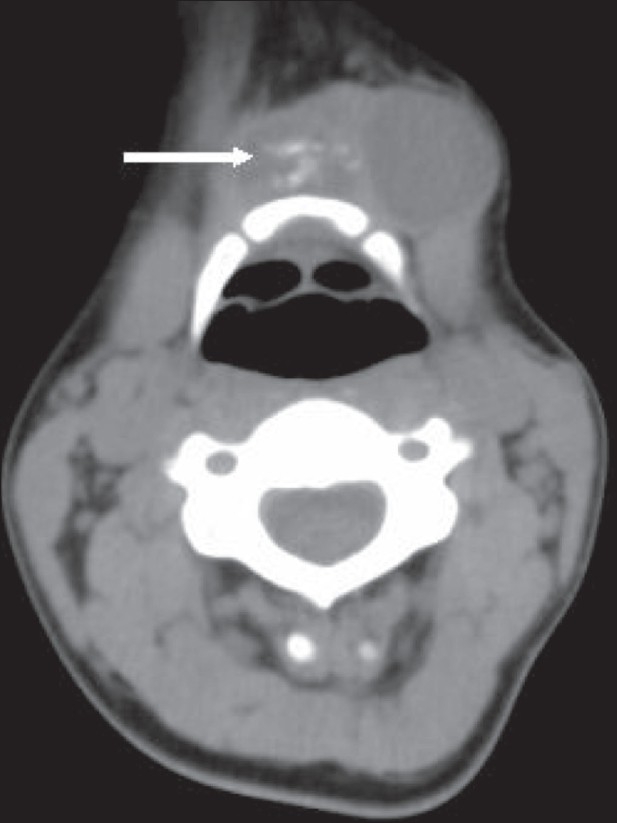
Axial non-contrast-enhanced CT scan at the level of the hyoid bone shows a multicystic lesion with calcification (arrow)

A 25-year-old woman presented with a left paramedian neck swelling that had been present for the last 3 years. It had increased in size over the last 3 months. There was no history of dysphagia, hoarseness, or fever. Physical examination revealed a firm, cystic swelling, measuring about 5 × 4 cm, which moved with deglutition and with protrusion of the tongue. Routine blood investigations and thyroid function tests were within normal limits. USG of the neck showed a well-defined, cystic mass in the left paramedian region at the level of the hyoid bone, with a heterogenous component in the submental region that showed calcification [Figure 1]. There was no significant cervical lymphadenopathy. The thyroid gland was normal. Fine needle aspiration cytology (FNAC) of the lesion was suspicious for mucoepidermoid carcinoma. To delineate the extent of the lesion, MRI was performed; it demonstrated a multicystic lesion (hypointense on T1W and hyperintense on T2W images) in the submental region, extending between the bellies of the mylohyoid muscles into the floor of the mouth and abutting the body of the hyoid. A few small hypointense foci were seen on T2W images that were suggestive of calcification [Figure 2a]. A large cystic component was seen in the left submandibular region, separate from the submandibular gland and in close relationship to the strap muscles [Figure 2b]. The smaller cysts in the submental region showed peripheral enhancement on CT and foci of calcification [Figure 3].

**Figure 4:**
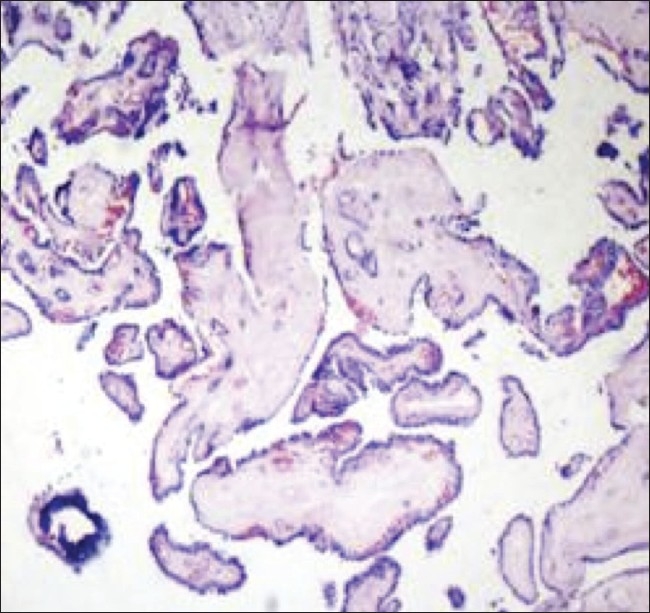
Low-power microscopy shows a cystic neoplasm composed of slender arborizing papillae with hyalinized fibrovascular cores lined by cuboidal cells, with nuclear crowding and overlapping. Psammoma bodies are seen

Based on the above imaging features, we felt that there was a strong possibility of a papillary carcinoma in a TDC. Sistrunk's procedure was performed and, per-operatively, a cystic mass with a solid component was noted close to the hyoid bone; it was adherent to the belly of the digastric muscle, the strap muscles, and the mylohyoid muscles. Histopathology of the resected specimen confirmed the diagnosis of a papillary carcinoma arising from a TDC [Figure 4].

## Discussion

Carcinoma in a TDC is seen in less than 1% of cases.[1] The first case was reported by Uchermann in 1915.[2] It occurs more commonly in women and is seen in the infrahyoid region along the course of the thyroglossal duct. The most common histological pattern demonstrated is that of papillary carcinoma of the thyroid.[3] Follicular variant of papillary carcinoma, pure follicular carcinoma, and squamous and anaplastic carcinomas are the less commonly encountered histological variants.

Being superficial, it is easily assessed by USG, which is the initial investigation of choice. On USG, a benign TDC can be anechoic, homogenously hypoechoic, homogenously hyperechoic (pseudo-solid), or heterogenous in appearance.[4] If calcification is seen within, malignancy needs to be suspected. In our case, apart from the cystic lesion, USG showed a heterogenous component with calcification. Calcification is the hallmark of papillary carcinoma in a TDC. To date, there have been no reports in the English language literature of calcification occurring in a benign TDC.[1]

The other possibilities to be considered during USG examination are dermoid cyst, epidermoid cyst, and synovial sarcoma. Epidermoid cysts rarely show calcification, while dermoid cysts may have internal echoes due to the presence of cholesterol crystals. Synovial sarcoma in the neck arises from the retrohyoid bursa and may be seen as a cystic lesion with calcification.[5]

The CT features of malignant TDC have been well documented. In a study by Barton *et al*., thyroglossal duct carcinomas were similar to benign TDCs in location; however, they showed a dense or enhancing mural nodule, calcification (60%), or both.[6] Taori *et al*. concluded that carcinoma should be considered in TDCs that have irregular calcification and high attenuation values.[1] In the present case too, CT scan showed high density (40–50 HU) within the cyst along with calcification. An infected TDC shows increased density and a thickened wall. Dermoid cysts may contain fat and are not closely related to the strap muscles. Calcification and the presence of a soft-tissue component are thus important CT features of malignancy in TDC. However, the eventual diagnosis will be based on histopathologic evaluation, either from a biopsy or after surgery.[7]

On MRI, a benign TDC may appear as a simple cyst (low T1 and high T2 signal intensity) or as a multilocular cystic lesion; however, it can have high T1 and T2 signal intensity, which is consistent with high protein content. Hemorrhage within a cyst may account for the variability of MRI intensity. Malignancy should be suspected if solid components are depicted in a TDC; however, inflammation can also lead to thickening of the cyst wall with solid components.[8]

The question still remains whether thyroid cancer in TDC is metastatic or is primary. The latter seems more likely, as normal thyroid tissue has been found to be present in two-thirds of thyroid remnants and a primary in the thyroid gland has not been detected in 50% of the cases.[9]

FNAC findings may be misleading in around 50% of cases due to the small size of the lesion or because of inadequate sampling due to hyperviscosity.[10] In our case, FNAC showed cells with vacuolated cytoplasm in a mucoid background, which were suspicious of mucoepidermoid carcinoma. Postoperative histopathology revealed a cystic neoplasm lined by cuboidal cells with nuclear crowding, intranuclear cytoplasmic inclusion, and moderate eosinophilic cytoplasm; there were also foci of squamous metaplasia and occasional psammoma bodies. A few ductules were seen extending into the surrounding muscle fibers. These findings are typical of papillary carcinoma.[11] Papillary carcinoma in a TDC has a good prognosis, and metastasis is reported to be exceedingly rare.[1]

We conclude that papillary carcinoma should be suspected in a TDC when there are imaging features such as calcification and the presence of soft tissue in a midline cystic mass, with a normal thyroid gland.
